# Radiolabeled Small Molecule Protein Kinase Inhibitors for Imaging with PET or SPECT

**DOI:** 10.3390/molecules15118260

**Published:** 2010-11-15

**Authors:** Justin W. Hicks, Henry F. VanBrocklin, Alan A. Wilson, Sylvain Houle, Neil Vasdev

**Affiliations:** 1 Department of Psychiatry, University of Toronto, 250 College St., Toronto, ON M5T 1R8, Canada; E-Mail: justin.hicks@utoronto.ca (J.W.H.); 2 Department of Radiology and Biomedical Imaging, University of California, San Francisco, 185 Berry St., San Francisco, CA 94107, USA; E-Mail: Henry.VanBrocklin@radiology.ucsf.edu (H.F.V); 3 PET Centre, Centre for Addiction and Mental Health, 250 College St., Toronto, ON M5T 1R8, Canada; E-Mails: salan.wilson@camhpet.ca (A.A.W.); sylvain.houle@camhpet.ca (S.H.)

**Keywords:** molecular imaging, protein kinase, small molecules inhibitors, positron emission tomography, single photon emission computer tomography

## Abstract

Imaging protein kinase expression with radiolabeled small molecule inhibitors has been actively pursued to monitor the clinical potential of targeted therapeutics and treatments as well as to determine kinase receptor density changes related to disease progression. The goal of the present review is to provide an overview of the breadth of radiolabeled small molecules that have been synthesized to target intracellular protein kinases, not only for imaging in oncology, but also for other areas of interest, particularly the central nervous system. Considerable radiotracer development has focused on imaging receptor tyrosine kinases of growth factors, protein kinases A, B and C, and glycogen synthase kinase–3β. Design considerations, structural attributes and relevant biological results are summarized.

## 1. Introduction

Protein kinases have emerged as one of the largest drug targets due to the pivotal role they play in signal transduction pathways. By extension, their dysregulation can cause significant alterations in many cellular processes, such as transcription, proliferation, angiogenesis, and inhibition of apoptosis, thereby contributing to a variety of illnesses including cancers and central nervous system (CNS) diseases [1]. Non-invasive detection of these changes can impact the choice of therapy as well as monitor the progress of those treatment decisions.

To date, the major focus of kinase drug development has been related to cancer treatment through targeted inhibition of overexpressed growth factors, with the aim of suppressing tumor growth [2]. Such inhibition has been attempted by either blocking the extracellular ligand binding domain with antibodies, affibodies or peptides, or by inhibition of the intracellular tyrosine kinase at the adenosine triphosphate (ATP) binding site with small molecule inhibitors [1,2,3]. There are four known monoclonal antibodies targeting the epidermal growth factor receptor (EGFR) or the vascular endothelial growth factor receptor (VEGFR), whereas fourteen small molecule tyrosine kinase inhibitors are approved for clinical use targeting EGFR, VEGFR, and other growth factor receptors [4]. Concomitantly, imaging the pharmacological destiny of these and other clinical agents by single photon emission computed tomography (SPECT) or positron emission tomography (PET) has been examined for applications in oncology and CNS research [4,5,6,7,8,9,10,11,12,13,14].

**Figure 1:**
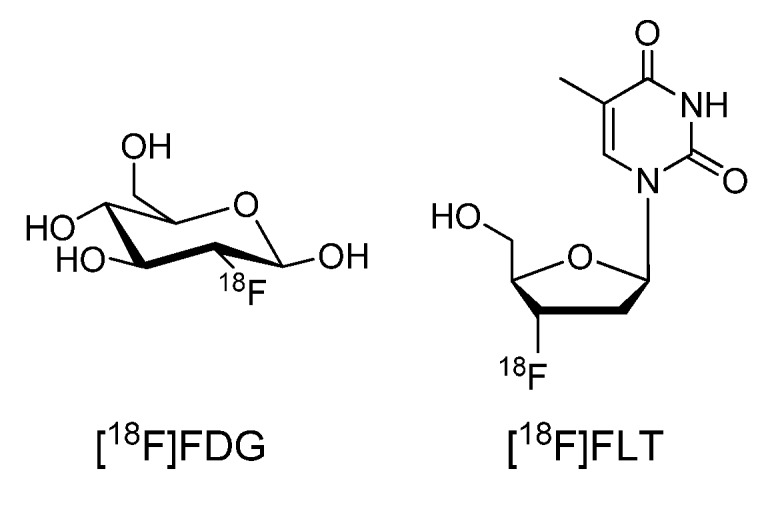
^18^F-labeled 2-fluoro-2-deoxy-D-glucose and 3'-deoxy-3'-fluorothymidine.

There are three common radionuclide-based molecular imaging approaches that have been used to monitor therapy and response of drugs targeting growth factor receptors: 1) indirect (surrogate) imaging of cellular metabolism and proliferation, 2) direct imaging of growth factor receptors with radiolabeled antibodies, affibodies, or peptides, or 3) imaging with radiolabeled small molecule protein kinase inhibitors. The most common and successful of these approaches has been indirect PET imaging with fluorine-18 labeled 2-fluoro-2-deoxy-D-glucose ([^18^F]FDG, Figure 1). Specifically, [^18^F]FDG is routinely used to detect glucose metabolism that is often increased in rapidly growing tumor tissues [15]. Following the administration of an effective kinase inhibitor as a chemotherapeutic agent, the tumor metabolism is expected to decrease with a consequent reduction in [^18^F]FDG uptake [16]. This approach has been applied to image the effects of targeted treatments of protein kinase inhibitors in cancer patients including monitoring the effects of Imatinib (Gleevec; STI571; Novartis) and Sunitinib (Sutent; SU11248; Pfizer) in patients with gastro-intestinal stromal tumors (GIST) [17,18]. A similar approach can also be used to measure cancer cell proliferation. Fluorine-18 labeled 3'-deoxy-3'-fluorothymidine ([^18^F]FLT, Figure 1) is a radiopharmaceutical used to interrogate DNA synthesis of growing cells [19], and it has been used to monitor the clinical efficacy of the small molecule inhibitor of EGFR tyrosine kinase, Gefitinib (Iressa; ZD1839; AstraZeneca) [20]. Although both [^18^F]FDG and [^18^F]FLT are capable of detecting early changes of biochemistry *in vivo*, these effects are considerably downstream from the signal initiation at the growth factor receptor. 

Imaging protein kinase expression with radiolabeled small molecule inhibitors may offer a direct and more sensitive approach to monitoring the clinical potential of targeted therapeutics and treatments. Thus, enormous potential exists for personalizing therapies, dose optimization, and monitoring therapeutic response. Furthermore, accurate quantification of kinase expression with radiolabeled kinase inhibitors could greatly accelerate drug discovery programs [21]. The goal of this review is to provide an overview of the structural diversity of radiolabeled small molecule protein kinase inhibitors that have been developed for imaging cancers and CNS disorders. Design consideration and relevant biological results are also presented.

## 2. Imaging Protein Kinase Expression with Small Molecule Radiolabeled Inhibitors for Cancer

**Figure 2:**
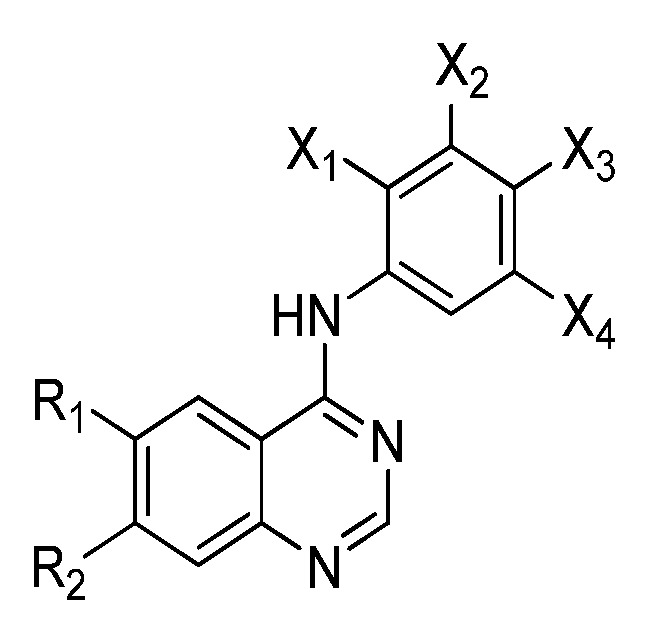
4-Anilino-quinazoline core of radiolabeled EGFR tyrosine kinase inhibitors (substituents listed in Table 1 and Table 2).

Efforts to image intracellular protein tyrosine kinases have focused on radiolabeling known drugs and related compounds that are in clinical trials. Characteristic examples are reversibly binding small molecule EGFR tyrosine kinase inhibitors. Compounds **1** and **2** are carbon-11 and fluorine-18 labeled isotopologues of Gefitinib, respectively [22,23,24,25], and **3** is [^11^C]-labeled Erlotinib (Tarveca; OSI-774; Genentech and OSI Pharmaceuticals) [26]. All of these compounds share a 4-anilino-quinazoline core and are based on the prototypical inhibitor, 6,7-dimethoxy-4-(3-bromoanilino)-quinazoline (PD153035). This inhibitor has been labeled with ^11^C in two different positions (isotopomers **4 **and **5**) [27,28,29,30], and recently **4 **advanced to preliminary human studies [31]. Several radiolabeled derivatives based on the 4-anilino-quinazoline core (Figure 2) have been prepared and this class of inhibitors can bind reversibly (Table 1) or irreversibly (Table 2) to the ATP binding domain of EGFR tyrosine kinase [32].

Beyond the isotopologues of clinically used reversible EGFR tyrosine kinase inhibitors (**1**–**5**), a number of rationally designed radiolabeled inhibitors for this target have been developed with a variety of isotopes and structural modifications. Preliminary reports describe the syntheses of two other reversibly binding [^11^C]-labeled 4-anilino-quinazoline derivatives (**6** and **7**) [33,34], however the majority of compounds in this class have been labeled with fluorine-18. The advantage of ^18^F over ^11^C is the longer half-life (t_½_ = 109.7 *vs.* 20.4 min, respectively), which may provide a better match of the pharmacokinetics of EGFR tyrosine kinase binding to the half-life of the tracer, in addition to potentially facilitating radiochemistry, allowing longer scanning times, and providing the opportunity to supply neighboring PET centers with the tracer. The 7-*O*-[^18^F]fluoroethyl derivative of PD153035 (**8**) was the first endeavor to prepare an [^18^F]-labeled reversible EGFR tyrosine kinase inhibitor [27], and modifications of [^18^F]-Gefitinib, compounds **9 **and **10,** were subsequently reported [25]. 

**Table 1:** Substituents of reversible radiolabeled EGFR tyrosine kinase inhibitors.

#	**R_1_**	**R_2_**	**X_1_**	**X_2_**	**X_3_**	**X_4_**	**Ref**
**1**	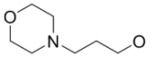	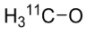	H	H	F	Cl	[22,23]
**2**	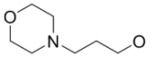	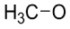	H	H	^18^F	Cl	[24,25]
**3**	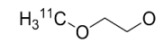	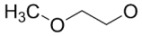	H	H	H	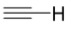	[26]
**4**	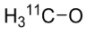	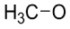	H	H	H	Br	[27,28,29]
**5**	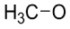	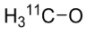	H	H	H	Br	[30]
**6**	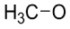	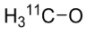	H	H	H	Cl	[33]
**7**	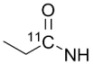	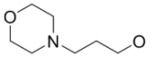	H	H	F	Cl	[34]
**8**	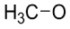	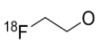	H	H	H	Br	[27]
**9**	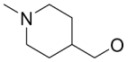	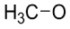	H	H	^18^F	Cl	[25]
**10**	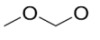	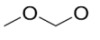	H	H	^18^F	Cl	[25]
**11**	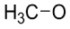	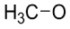	H	H	^18^F	Cl	[35]
**12**	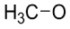	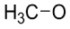	^18^F	H	Cl	Cl	[36]
**13**	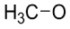	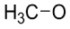	H	CF_3_	H	^18^F	[36]
**14**	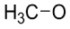	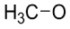	H	H	^18^F	H	[36,37]
**15**	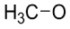	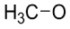	H	^18^F	H	H	[37]
**16**	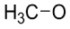	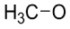	^18^F	H	H	H	[37]
**17**	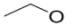	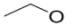	H	H	^18^F	H	[37]
**18**	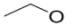	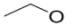	H	^18^F	H	H	[37]
**19**	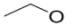	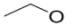	^18^F	H	H	H	[37]
**20**	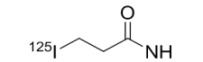	H	H	H	F	Cl	[38]
**21**	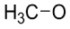	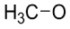	H	H	H	^125^I	[39]
**22**	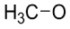	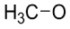	H	H	H	^123^I	[40]
**23**	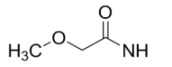	H	H	H	H	^124^I	[41]
**24**	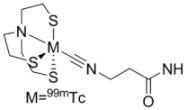	H	H	H	F	Cl	[42]
**25**	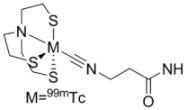	H	H	H	H	Br	[42]
**26**	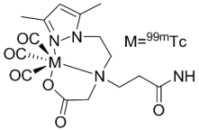	H	H	H	F	Cl	[42]

**Table 2:** Substituents of irreversible radiolabeled EGFR-tyrosine kinase inhibitors.

#	R_1_	R_2_	X_1_	X_2_	X_3_	X_4_	Ref
**27**	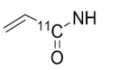	H	F	H	Cl	Cl	[43]
**28**	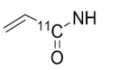	H	H	H	H	Br	[43]
**29**	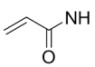	H	^18^F	H	H	H	[44]
**30**	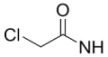	H	^18^F	H	H	H	[45]
**31**	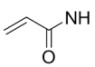	H	H	H	H	(CH_2_)_2_[^18^F]	[46]
**32**	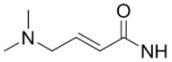	H	^18^F	H	Cl	Cl	[47]
**33**	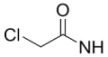	H	H	H	H	^124^I	[41]
**34**	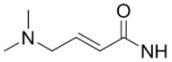	H	H	H	H	^124^I	[41]
**35**	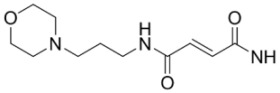	H	H	H	H	^124^I	[48]
**36**	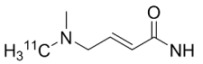	H	F	H	Cl	Cl	[49]
**37**	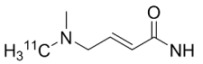	H	H	H	H	I	[49]
**38**	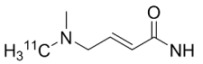	H	H	H	H	Br	[49]
**39**	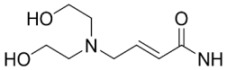	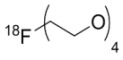	F	H	Cl	Cl	[50]
**40**	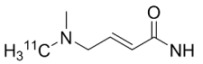	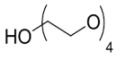	F	H	Cl	Cl	[50,51]
**41**	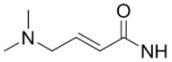	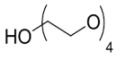	H	H	H	^124^I	[50,51]
**42**	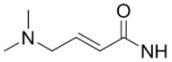	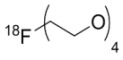	F	H	Cl	Cl	[51,52]
**43**	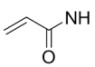	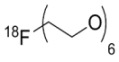	H	H	H	I	[53]
**44**	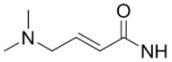	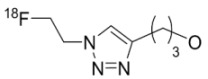	H	H	F	Cl	[54]

Fluorine-18 labeled derivatives of PD153035, **11–19**, have also been synthesized where the number and position of halogens on the [^18^F]fluoroanilino group have been varied [35,36,37]. Compounds **12**–**14** were evaluated by *in vitro* and *in vivo* studies in tumor bearing mice [36]. While *in vitro* experiments were promising, kinetic factors and rapid blood clearance deemed these compounds to be unsuitable for imaging EGFR tyrosine kinase *in vivo*. Compounds **14**–**19 **were prepared to systematically evaluate the biological influence of fluorine at the 2-, 3- or 4-positions on the anilino moiety [37]. *In vitro* evaluation of these compounds in human female hepatocytes revealed rapid degradation of 6,7-dialkoxy-4-(4-[^18^F]fluoroanilino)-quinazolines (**14** and **17**) [55]. This was corroborated *in vivo* by high bone uptake, consistent with defluorination and previously reported decomposition of para-fluoroanilines [35]. Thereby, the 2- and 3-[^18^F]fluoroanilino derivatives are likely to have more suitable metabolic properties for *in vivo* imaging. It is noteworthy that two isotopomers of [^11^C]PD153035 (**4 **and **5**) were evaluated in human and rat microsomes, and the 7-*O*-[^11^C]methoxy derivative (**5**) was more desirable because its radiometabolites were polar and likely cleared rapidly [30]. In light of this study, it is surprising that **4** was selected for human evaluations [31]. These studies illustrate the importance of evaluating isotopomers of radiotracers in radiopharmaceutical design and preclinical studies.

The timeframe to access EGFR tyrosine kinase may not be suitable using radiotracers with short-lived isotopes, such as ^11^C or ^18^F. Reversible inhibitors of EGFR tyrosine kinase labeled with isotopes of iodine (**20–23**) [38,39,40,41] or ^99m^Tc (**24–26**) [42] were designed to be longer-lived radiotracers. Although longer half-lives (^123^I, t_½_ = 13.3 h, ^124^I, t_½_ = 100.2 h, ^125^I, t_½_ = 59.6 d; ^99m^Tc t_½_ = 6.02 h) may give better distribution kinetics, the increase in lipohilicity and size of incorporating iodine or metal chelates into the quinazoline scaffold likely hindered the retention of these compounds into the binding pocket of EGFR tyrosine kinase. Another factor which is detrimental to all of the compounds in Table 1 is the competition between high levels of intracellular ATP with the reversible inhibitors for occupying the tyrosine kinase domain. Compounds in Table 2 were developed to overcome this competition by including a functional group at position 6 (R_1_, Figure 2) of the same quinazoline core which can irreversibly bind to cysteine 773 of the EGFR tyrosine kinase [8].

**Figure 3:**
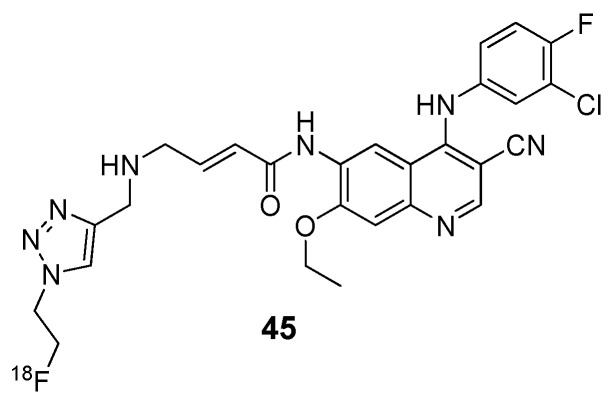
Recent click chemistry to form a [^18^F]-labeled EGFR tyrosine kinase targeting probe.

The first report on irreversibly binding EGFR tyrosine kinase inhibitors labeled with ^11^C (**27 **and **28**) was by Mishani and coworkers [43] and the first compounds in this class labeled with ^18^F (**29 **and **30**) were prepared in our laboratories [44,45]. Other efforts to develop an irreversibly binding EGFR tyrosine kinase probe include quinazoline derivatives labeled at the [^18^F]fluoroanilino (**31** and **32**) [46,47] or [^124^I]iodoanilino moieties (**33–35**) [41,48], as well as [^11^C]-labeled groups at position 6 (**36**–**38**) [49]. Attempts to increase the hydrophilicity of irreversibly binding radiotracers by incorporating polyethylene glycol (PEG) side-chains (**39**–**43**) initially appeared promising [50,51,52] but recent biological evaluations of compounds **40**–**42** showed little improvement on specific binding and tumor uptake in animal models [51]. Exploration of click chemistry in this class of compounds is also underway (compound **44**) [54] with a recent report also exploring a slight modification to this scaffold with a 4-anilino-3-cyanoquinoline core (**45**, Figure 3) [56].

Many of the compounds in Table 2 have undergone *in vitro* and *in vivo* experiments to determine their suitability for imaging EGFR tyrosine kinase. Similar to the reversible compounds, the majority of *in vitro* assays showed very promising pharmacological profiles for imaging; however subsequent *in vivo* studies were generally unfavorable. To date all of the small molecule radiotracers that target EGFR tyrosine kinase have shown low specific uptake into tumors and/or insufficient signal to noise for clinical imaging [9]. Several factors have been rationalized for the lack of success with radiolabeled 4-anilino-quinazolines for imaging EGFR tyrosine kinase *in vivo *and include problematic metabolism, insufficient cellular penetration, and relatively high lipophilicity that contributes to high non-specific binding. Although significant improvements can be made to the small molecule inhibitors, radiolabeled antibodies, affibodies and peptides that bind to the extracellular domain of EGFR have been more successful for imaging this target [4,5,6,7,8,9,10,11,12,13,14].

**Figure 4:**
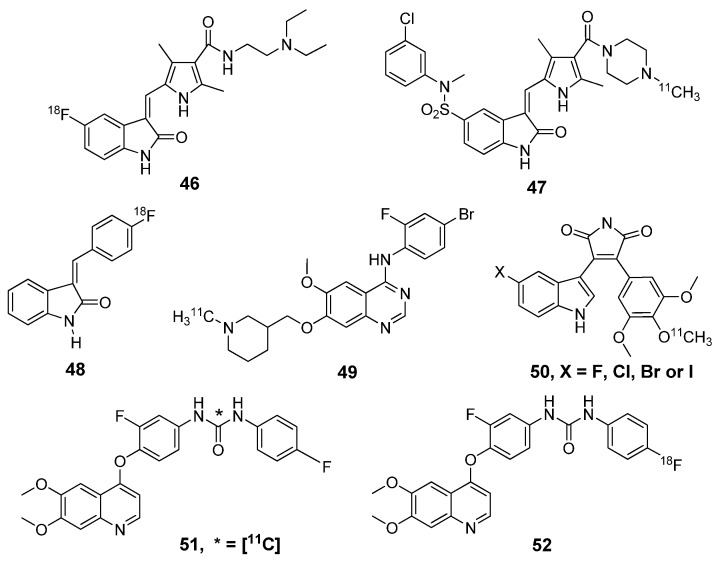
Radiolabeled VEGFR tyrosine kinase inhibitors.

Apart from EGFR, there are several other growth factor receptors of interest in cancer therapy. For example, overexpression of VEGFR, responsible for the vascularization of tumors, can result in sustained rapid growth and migration through the circulatory system [57]. Determining the location and density of these receptors by molecular imaging could assist in the therapy of patients with VEGFR positive tumors. Examples of radiolabeled small molecule VEGFR tyrosine kinase inhibitors are shown in Figure 4. It is no surprise that efforts to design VEGFR tyrosine kinase radiotracers have also pursued the labeling of known inhibitors or their analogs. Recently, Sunitinib and two derivatives have been radiolabeled with ^11^C or ^18^F (**46**–**48**, Figure 4) and their suitability for imaging VEGFR are being explored [58,59,60].

Other recent efforts employing the 4-anilino-quinazoline scaffold (characteristic of EGFR tyrosine kinase inhibitors; *vide supra*) include the development of compound **49**, and tumor uptake correlated well to various levels of VEGFR tyrosine kinase expression in different xenograft models [61]. Other [^11^C]-labeled compounds for this target, based upon an indole-maleimide core, were prepared (**50)** [62], as well as a dual receptor probe for VEGF/platelet-derived growth factor tyrosine kinase, labeled with ^11^C or ^18^F (**51** and **52**, respectively) [63,64]. Similar to radiotracers developed for EGFR tyrosine kinase, radiotracers targeting VEGFR tyrosine kinase have not proved useful for clinical applications despite promising *in vitro* evaluations and may benefit from systematic studies to determine optimal properties for imaging this target.

**Figure 5:**
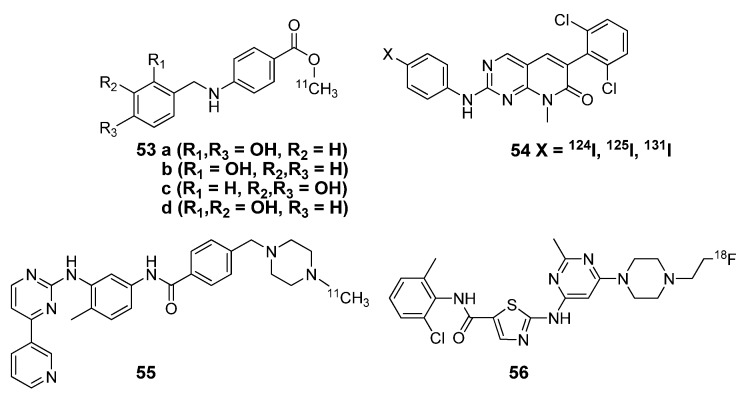
Radiotracers of other kinase inhibitors for cancer imaging.

Small molecule radiotracers under development for cancer imaging extend beyond targeting receptor tyrosine kinases and include other oncogenic kinases, such as Abl or cKit. The first radiolabeled compounds attempting to target Bcr-Abl kinase (notably upregulated in chronic myelogenous leukemia, CML) are [^11^C]AG957 and its derivatives (**53**, Figure 5) but the selectivity of these compounds are insufficient for imaging [65]. Early work beyond this probe was conducted by labeling the Bcr-Abl inhibitor SKI-230 (**54**) with various radioisotopes of iodine [66] and a recent report showed favorable *in vivo* results as an Abl radiotracer [67]. Imatinib, a drug that targets c-Kit, as well as Bcr-Abl kinase, is approved for clinical treatment of CML and GIST. Because many of the cancer patients treated with Imatinib developed a drug resistance, new drugs, such as Dasatinib **(**Sprycel; BMS-354825; Bristol Meyer Squibbs), were prepared to improve its properties and respective radiotracers followed suit. Carbon-11 labeled Imatinib (**55**) [68] and a [^18^F]fluoroethyl derivative of Dasatinib (**56**) [69] have been prepared and evaluated by *in vivo* animal studies. Although these radiotracers have potential for imaging oncogenic kinases, further validation is required in human cancer patients. 

**Figure 6:**
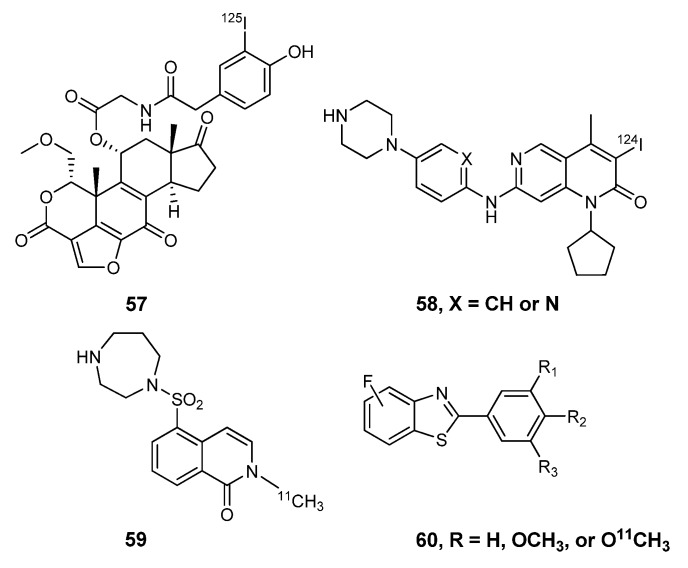
Radiolabeled small molecule inhibitors of multifunctional protein kinases.

Radiolabeled small molecule kinase inhibitors have also been developed to target multifunctional protein kinases. Phosphatidylinositol 3-kinase (PI3K) is involved in multiple signal transduction pathways, and a derivative of Wortmannin (a fungal product) labeled with ^125^I (**57**, Figure 6) was explored for studying this target [70]. The cyclin-dependent kinase (CDK) family of multifunctional protein kinases contributes to the regulation of the cell cycle. With the goal of imaging the expression of CDK4, [^124^I]-labeled compounds were developed (**58**), but unfortunately these had poor *in vivo* stability and consequently low tumor uptake in mice [71]. Recently, *N*-[^11^C]methyl-hydroxyfasudil (59) was reported as a potential radiolabeled Rho-kinase (ROCK; a multifunctional protein kinase) inhibitor with the goal of cardiac imaging [72]. Finally, a series of fluoro-arylthiazoles have been labeled in a variety of positions with carbon-11 (**60**) and are under exploration for imaging EGFR and Abl tyrosine kinases [73]. 

Despite extensive research to develop small molecule radiotracers to image oncogenic kinases, only one radiotracer has translated to preliminary human imaging studies [31]. Significant efforts have been directed towards increasing the affinity of these probes towards protein kinases; however, limited work has focused on determining the specificity of compounds across a broad spectrum of kinases. Moreover, rationally designed *in vivo* imaging studies, in conjunction with *ex vivo* biodistribution and pharmacological evaluations in appropriate xenograft models may accelerate development of radiopharmaceuticals targeting protein kinases. Fundamental research is required to systematically improve the pharmacological properties of lead compounds for routine clinical use. 

## 3. Radiolabeled Small Molecule Protein Kinase Inhibitors for Imaging in the CNS

When imaging the expression of protein kinases in the brain, surrogate methods (as previously described in cancer imaging) can also be used to image the effects of a protein kinase inhibitor on a secondary process. For example, the effects of a protein kinase A (cyclic adenosine 3',5'- monophosphate (cAMP)-dependent protein kinase) inhibitor were evaluated by small animal imaging with [^18^F]FDG [74]. Furthermore, [^11^C] and [^18^F]forskolin were employed to quantify cAMP levels [75,76]. Unfortunately, there are very few examples of intracellular kinase targeting radiotracers which focus on the CNS, whereas compounds that inhibit these targets are highly pursued in drug discovery programs. There are many kinases implicated in neuropathologies [77,78] that include PI3K, CDKs, protein kinase A, B and C (PKA, PKB and PKC, respectively), as well as glycogen synthase kinase-3β (GSK-3β). Limited attempts to image such protein kinase targets in the brain with radiolabeled small molecules in our laboratory and others are outlined below. 

### 3.1. Protein kinase A, B and C

**Figure 7:**
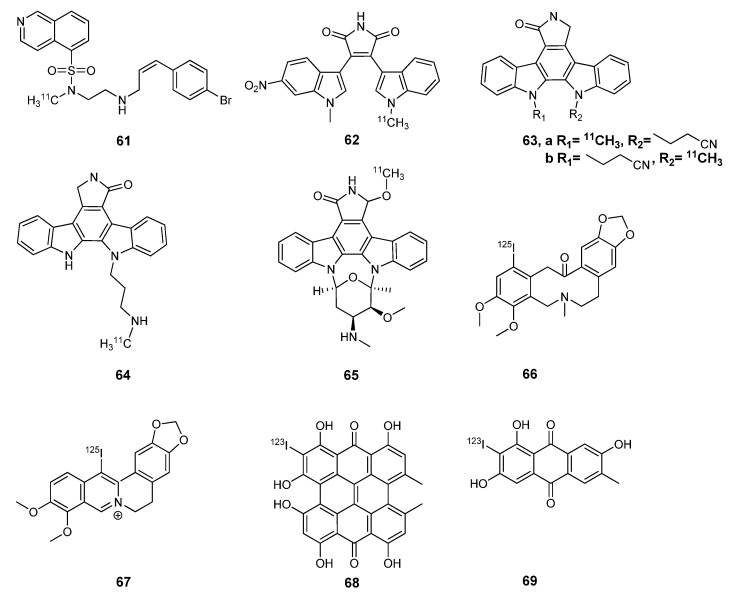
Radiolabeled small molecule inhibitors of protein kinases with potential CNS applications.

Protein kinase A is a mediator of the cellular response to cAMP, a secondary messenger utilized by both hormones and transmitters, and its dysregulation has been linked to depression [79,80]. There is only one report attempting to directly image PKA, using carbon-11 labeled *N*-(2-(4-bromocinnamylamino)ethyl)-*N*-methyl-isoquinoline-5-sulfonamide, an *N*-methyl derivative of the known PKA inhibitor, H89 (**61**, Figure 7) [81]. Unfortunately, this compound has poor brain penetration and cannot be used to image PKA expression in the CNS. While PKB (also known as Akt) is more frequently associated with oncology, it can have multiple functions in different pathways, including interacting with PI3K and GSK-3β in the CNS [82]. Recently, a [^11^C]bisarylmaleimide **62 **was reported for imaging PKB and further biological evaluations will determine its utility [83]. Preliminary work has further explored [^11^C]bisarylmaleimides for imaging PKC (**63** and **64**) [84,85]. Protein kinase C (calcium/calmodulin-dependent kinase) is also part of the PI3K signaling pathway and plays a crucial role in intracellular signal transduction [86]. Early work to develop a PET radiotracer for PKC began by labeling a staurosporine derivative with carbon-11 [87]. While staurosporine itself lacks specificity required for imaging, the 7β-methoxy derivative **65** is 57-fold more selective towards PKC with an IC_50_ value of 1.4 nM. Unfortunately, the radiochemical yield was too low to enable further biological evaluations with this tracer. Compounds **66 **and **67** were both labeled with the gamma-emitting [^125^I] *via* isotope exchange and both were fairly stable *in vivo *[88,89]. Compound **67** was evaluated in a biodistribution study, and not surprisingly the brain was excluded as this compound is charged and unlikely to penetrate the blood brain barrier [89]. Preliminary proceedings have been reported on two promising ^123^I-labeled compounds derived from St. John’s wort (*Hypericum perforatum*) as potential imaging agents for PKC (**68** and **69)** [90,91,92]. The development of a radiotracer suitable for imaging PKA, PKB, or PKC remains an ongoing goal in several laboratories.

### 3.2. Glycogen synthase kinase-3β

**Figure 8:**
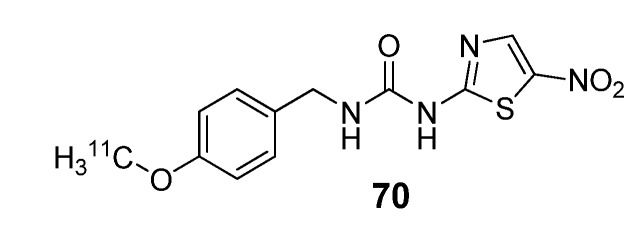
*N*-(4-[^11^C]methoxy)-*N*'-(5-nitro-1,3-thiazole-2-yl)urea ([^11^C]*methoxy*-AR-A014418).

**Figure 9:**
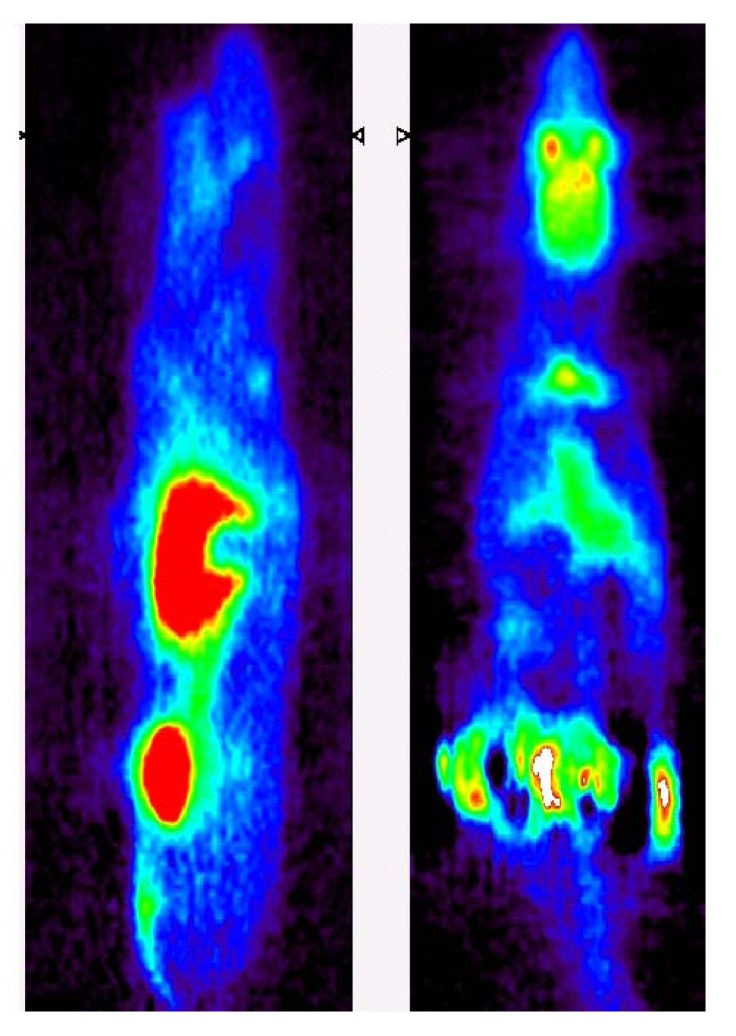
Representative *in vivo* PET images following intravenous administration of **70** (left) or [^18^F]FDG (right) in Sprague-Dawley rats [98].

Glycogen synthase kinase-3β is a serine/threonine kinase that is highly abundant in brain tissues and involved in signal transduction cascades of multiple cellular processes. Small molecule inhibitors of GSK-3β are currently under development for a broad range of illnesses including depression, diabetes, stroke, bipolar disorder, malignancy [93], and most recently a ‘GSK-3 hypothesis of Alzheimer’s disease’ has been proposed [94]. One potent and selective inhibitor for this target is *N*-(4-methoxy)-*N*'-(5-nitro-1,3-thiazole-2-yl)urea (AR-A014418; AstraZeneca) [95]. This compound was labeled with carbon-11 at the methoxy-position (70; Figure 8), however, *ex vivo* biodistribution showed that there was no appreciable brain uptake of this radiotracer following injection in conscious rodents, despite the pre-administration of a P-glycoprotein inhibitor [96]. This lack of brain uptake was readily visualized by *in vivo* imaging in rodents, using a human PET scanner (High Resolution Research Tomograph, CPS Innovations) [97] where two rats were simultaneously imaged under anesthesia with either [^11^C]*methoxy*-AR-A014418 or [^18^F]FDG (Figure 9) [98]. Similarly, no brain uptake was seen when imaging [^11^C]*methoxy*-AR-A014418 in porcine models (Dr. Steen Jacobsen, personal communication). 

Recent work has focused on systematic derivatization of AR-A014418 and *in vitro* screening of the new compounds, in conjunction with X-ray crystallographic studies, with the goal of developing new inhibitors and radiopharmaceuticals for imaging GSK-3β [99,100]. Carbon-11 labeled *methoxy*-AR-A014418 was previously prepared by methylating the phenolic oxygen with [^11^C]methyl iodide, via the “Loop” method [101,102]. Although this methodology is highly efficient and established, the chemistry is restrictive to reactions with [^11^C]methyl iodide, or a related methylating reagent such as [^11^C]methyl triflate. Newly developed [^11^C]CO_2_ fixation chemistry [103,104,105] can be applied to prepare a variety of [^11^C]ureas labeled in the carbonyl position. This approach will enable the preparation of large arrays of urea-based radiotracers within a compound class, such as AR-A014418 derivatives, and may prove useful in development of radiolabeled small molecule kinase inhibitors. 

## 4. Conclusions

Development of small molecules that inhibit protein kinases *in vivo* are currently one of the most aggressively pursued areas of drug discovery as there are many kinases that are implicated in the pathology of cancers, cardiac, and CNS diseases, yet this field is in its infancy in molecular imaging. The most promising work to date has been accomplished by imaging of these targets with surrogate tracers (i.e. [^18^F]FDG or [^18^F]FLT), or by use of large molecules (antibodies, affibodies and peptides) that target the extracellular domain of the receptors. The development of small molecule radiotracers to image protein kinases is a particularly challenging goal as the difficulties of intracellular imaging are compounded by obtaining adequate potency and selectivity over closely related kinases. This presents additional burdens to the already considerable obstacles to develop any radiopharmaceutical [106,107]. Moreover, the development of such radiotracers to target the CNS is further hindered by the challenges of surpassing the blood-brain barrier. More efforts need to be directed towards systematic studies to discovering and screening lead compounds for these high risk (albeit high gain) targets. Such is the dire need for new targeted radiotracers that can non-invasively and accurately quantify protein kinases *in vivo* for not only guiding drug translation, but also for offering new diagnostic and therapeutic imaging options to patients, that efforts in this challenging area must be encouraged.
